# A Definition of “Regular Meals” Driven by Dietary Quality Supports a Pragmatic Schedule

**DOI:** 10.3390/nu12092667

**Published:** 2020-09-01

**Authors:** Barbara Lohse, Kathryn Faulring, Diane C. Mitchell, Leslie Cunningham-Sabo

**Affiliations:** 1 Wegmans School of Health and Nutrition, Rochester Institute of Technology, Rochester, NY 14623, USA; kzfihst@rit.edu; 2 Department of Nutritional Sciences, The Pennsylvania State University, Centre County, PA 16801, USA; dcm1@psu.edu; 3 Department of Food Science and Human Nutrition, Colorado State University, Fort Collins, CO 80526, USA; Leslie.Cunningham-Sabo@Colostate.edu

**Keywords:** meal timing, dietary patterns, chrononutrition, dietary quality, eating competence, dietary assessment

## Abstract

Public health guidelines advise eating regular meals without defining “regular.” This study constructed a meaning for “regular” meals congruent with dietary quality. Parents of 4th grade youth in a school-based intervention (Clinicaltrials.gov NCT02491294) completed three, ASA24 online 24-h dietary recalls. Differences in time of intake across days for breakfasts, lunches, dinners were categorized with consistency denoted as always, often/sometimes or rarely/never and assigned values of 3, 2 or 1, respectively. Meal-specific values were summed to form mealtime regularity scores (mReg) ranging from 3 (low) to 9. Healthy eating index (HEI) scores were compared to mReg controlling for weekday/weekend recall pattern. Linear regression predicted HEI scores from mReg. Parents (*n* = 142) were non-Hispanic white (92%), female (88%) and educated (73%). One mReg version, mReg1 was significantly associated with total HEI, total fruit, whole fruit, tended to correlate with total protein, seafood/plant protein subcomponents. mReg1 predicted total HEI (*p* = 0.001) and was inversely related to BMI (*p* = 0.04). A score of three (always) was awarded to breakfasts, lunches or dinners with day-to-day differences of 0–60 min; also, lunches/dinners with one interval of 60–120 min when two meals were ≤60 min apart. More rigid mReg versions were not associated with dietary quality.

## 1. Introduction

The value placed on eating regular meals is evidenced in population health, dietary guidance and inclusion in scientific research. For example, the National Health and Nutrition Examination Survey (NHANES) includes questions about timing of meals [1]; weight loss programs advise clients to eat regular meals [2]; dietary guidelines of some countries focus on regular meals, e.g., Japan’s advice to “Establish a healthy rhythm by keeping regular hours for meals,” [3]; and the healthy lifestyle index includes eating regular meals and applies these findings to healthy aging policies [4]. Attention to eating regular meals has stemmed from research associating regular meals with lower incidence of overweight and obesity in youth [5,6] and adults [7]; more protection against youth substance abuse [8] and oppositional behavior [9], metabolic syndrome [10]; and favoring development of a healthy cardiometabolic profile [11]. The medical importance of regular meals has been applied to lower incidence of gastroesophageal reflux [12] and chronic uninvestigated dyspepsia [13], modulation of the microbiome [14], biomarkers of oxidative stress [14,15] and medication bioavailability [16]. Discoveries about the impact of cellular circadian clocks on health [14,17,18], the contribution of genetic variation to breakfast skipping [19] and the modulation of meal frequencies on SNPs associated with obesity risk [20] add credence to focus on regular meals.

These relationships have been promulgated by nutritionists without a standard definition of “regular meals.” Research on family meal frequency has led to categorizing family meals as never, 1–2 times, 3–6 times and seven or more times each week [21], then declaring three or more family meals a week as “regular” [8]. A measure of family regularity has been calculated from family routines (e.g., bedtime) that included mealtime frequency ascertained only from reported numbers of meals during a week. The resulting index was applied to behavioral measures of youth oppositional behavior [9]. In addition to frequency, the word “regularity” may be included in questions exploring mealtime patterns. For example, asking how often meals are consumed regularly (with options of never, occasionally, often or always) [12]. Another definition applied to a regular meal specifically for children, was eating a meal on every school day; a regular meal pattern was usually eating breakfast, school lunch and dinner on each school day [6]. Establishing a definition for mealtime regularity highlights the question of how “meals” themselves are defined [22] and differentiated from snacks since most studies use a participant-defined approach [23]. The American Heart Association suggests letting participants distinguish between meals and snacks to accommodate social norms and cultures, but that researchers define meals as providing ≥15% of total energy intake and snacks less than that with at least 15 min between separate eating occasions [11].

The purported relationships between mealtime regularity and health and have been challenged because of an absence of a standardized definition of mealtime regularity and the limited evidence on the basis for the most commonly used definitions. For example, Miller et al. [24] did not find beneficial effects of family meal frequency on child behavioral or academic outcomes when they examined breakfast and dinner separately and analyzed continuous frequencies, rather than categorized values. BMI was not shown to be related to regularity of eating occasions when compared to a chaotic eating index based on meal timings [25].

How to classify eating episodes has been a concern for decades [26]. The purpose of this study was to contribute to the development of a standard definition for mealtime regularity by comparing the consistency of timing of meals across three 24-h recalls with dietary quality and BMI.

## 2. Materials and Methods

### 2.1. Study Design

This project was a secondary analysis using parent dietary records that were a component of a cluster randomized study of the impact of a culinary and physical intervention, *Fuel for Fun (FFF)*, delivered during the year their child was in 4th grade [27]. Although the primary study included data collection at up to 4 time points, the only dietary records included for this study were those for the first time dietary data were collected. This was either at baseline or, if dietary records were not completed during the time their child was in *FFF*, it was from a dietary assessment completed at the time of the longitudinal follow-up. The longitudinal follow-up ranged from two to five years after initial participation, depending on the cohort of their child’s participation.

### 2.2. Recruitment

Parents of youth whose schools and classrooms were participating in *FFF* were recruited through flyers and announcements that encouraged access to a link that provided informed consent and a survey set of questions related to dietary and mealtime behaviors and demographic items. At the end of the survey, an item recorded interest in participating in a dietary assessment component. Affirmative responses triggered the informed consent for the dietary assessment; consenting participants’ contact information was forwarded to the Diet Assessment Center at The Pennsylvania State University who coordinated the collection and analysis of the dietary assessments. For the longitudinal follow-up, contact information previously collected from participating parents enabled emailing parents about the follow-up study with a link to another informed consent and a survey set that included a question of interest in the dietary collection component and an informed consent for those who were interested. For this follow-up component, dietary collection was coordinated by the authors using the methods comparable to those of the diet assessment center. Inclusion criteria for this study consisted of being a previous parent participant in the *Fuel for Fun* impact study and completion of three 24-h recalls at either the baseline of the primary study or of the longitudinal study. Parents who completed these recalls at both time points were included as test-retest participants.

### 2.3. Data Collection

Demographic and eating behavior data were collected using an online survey platform (Qualtrics, Provo, UT, USA). Dietary data were collected from 24-h recalls using the National Cancer Institute online automated self-assessment 24-h (ASA24) dietary assessment tool [28]. In addition to nutrient and food group data, information is collected on time and location of consumption. For participants completing only a baseline dietary assessment or a longitudinal follow-up assessment the average number of days between first and third recall was 11.7 ± 7.9 days (median 9 days; range 3–41 days). For those completing a second dietary assessment, which occurred during the longitudinal follow-up, the average number of days between first and third recall of this second assessment was 10.6 ± 7.1 days (median 8.5 days; range 4–38 days). The protocol to capture dietary data consisted of sending an unannounced email to a participant requesting completion of a dietary recall for the previous day. Emails were continued until three recalls was completed. Invitations were scheduled to attempt to include two weekdays and one weekend day. Participation incentives consisted of $10–20 per online survey set and $10–15, $15–20 and $15–25 for the first, second and third ASA24 recalls, respectively, depending on study cohort with later cohorts receiving the larger incentive.

### 2.4. Instruments

In addition to the ASA24 online dietary assessment tool, the survey set on the Qualtrics platform included validated surveys and tested items to examine participant eating behaviors, mealtime attitudes and practices toward their eating and family meals, as well as demographic information including food security and stress. Eating competence was measured with the Satter Eating Competence Inventory (ecSI 2.0^TM^). Construct validity was identified for this 16-item tool [29,30,31] that includes 5 response options for each item. Possible scores are 0 (low eating competence) to 48 (fully eating competent) because two of the response options (never, rarely) are both scored zero. Scores of 32 or more are indicative of being eating competent. The ecSI 2.0^TM^ consists of four subscales that reflect eating competence tenets: eating attitudes (6 items); internal regulation (2 items); food acceptance (3 items); and contextual skills (5 items) [31]. Cronbach’s alpha for this sample was 0.90.

Parent feeding behaviors were categorized with the caregiver’s feeding style questionnaire (CFSQ) [32]. This scale identifies four parent feeding styles: Indulgent, Uninvolved, Authoritarian and Authoritative. The five response options for each of the 19 items were summed and averaged to determine levels of parent demandingness and responsiveness. Cut points for low-income samples were applied to reveal the parent feeding style.

Parent perceived stress was assessed using a visual analog scale for one item that is anchored by 1 (no stress) and 10 (extreme stress). This item was previously tested in the Community Health Database [33]. In addition to demographics on age, sex, ethnicity, education, family size and participation in assistance programs, practices and feelings related to food preparation (e.g., frequency of food preparation, amount of time spent on food preparation) and worry about money for food were captured by several items tested for comprehension and used in several studies [34]. Height and weight were self-reported.

### 2.5. Data Analyses

#### Development of Mealtime Regularity Score (mReg)

**Figure 1:**
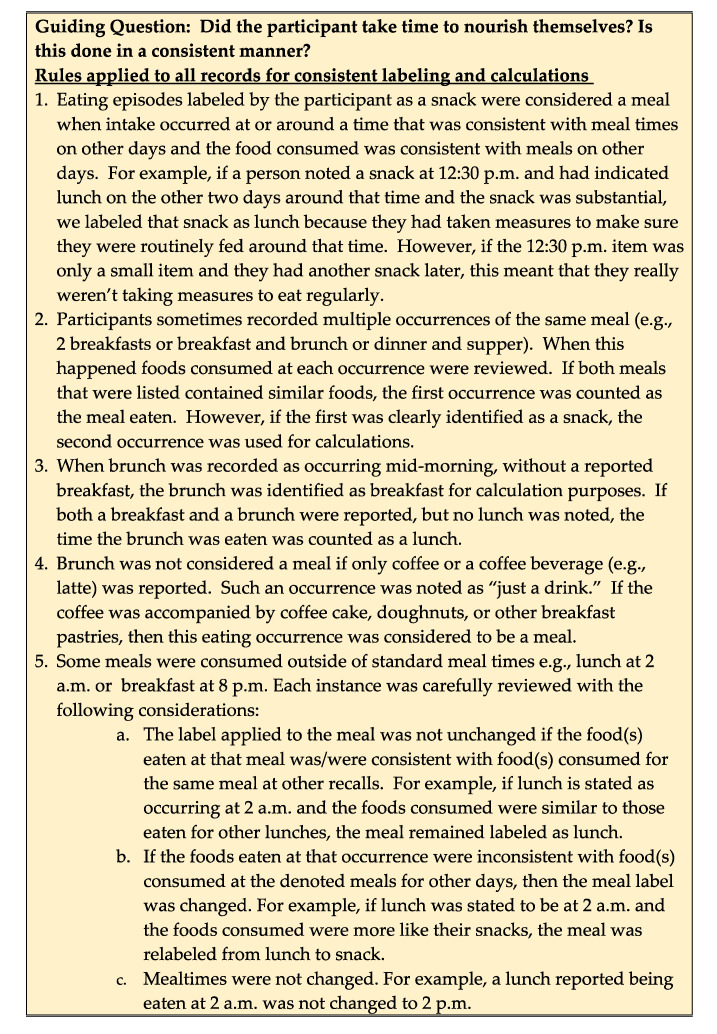
Guidelines for labeling meals and intervals.

A score to indicate the regularity of participant mealtimes was developed in four steps:

1. For each meal, the time interval between first, second and third recalls was calculated and recorded. For nearly all participants, listing of meals and times was straightforward, e.g., recall 1 breakfast 8:00 a.m., recall 2 breakfast 8:30 a.m.; and recall 3 breakfast 3 8:30 a.m. However, a small number of participants provided unclear or conflicting entries, e.g., Recall 1 breakfast 6 a.m., Brunch 9 a.m., Lunch 12 p.m. For these and other ambiguous entries, a decision guide was developed to assure uniform decisions were applied across similar situations (Figure 1).
2. An interval-driven scoring system was developed that provided higher scores to smaller intervals, i.e., greater regularity. One, two or three points were allocated to descriptors of rarely/never, often/sometimes and always, respectively. In all, three scoring schemes were developed. The first scoring scheme (version 1) allocated points based on meal-specific day-to-day intervals derived from informal interviews with project personnel about mealtime practices. This scheme resulted in a disproportionately high number of always designations for lunch and dinner. To facilitate a more proportionate distribution among the descriptors, a second scoring algorithm (version 2) was developed that more narrowly defined the descriptors for meals. For example, in version one, an interval of 0 to 60 min between the three breakfasts was denoted as always; this was changed to 0 to 30 min in version 2. However, several breakfast intervals were just a few minutes over the 30 min cutoff for version 2 breakfast, prompting concern that version 2 was too rigid for breakfast. Therefore, a third scoring version was included that differed from version 2 only in having a slightly expanded interval for breakfast to be denoted as always regular. The frequency-driven descriptors of mealtime intervals for the three temporally specific scoring versions are detailed in Table 1.
3. For each scoring version, based on the descriptor applied to the day-to-day time difference for each meal, participants received a score (from 1 to 3) each for breakfast, lunch and dinner. Meal-specific regularity scores were summed to produce a mealtime regularity score for each version (i.e., mReg1, mReg2, mReg3) that could range from 3 (least regular) to 9 (most regular).

The healthy eating index (HEI) and subcomponent scores were calculated from ASA24 data using the 2010 Dietary Guidelines for Americans (most current version when data collection began) [35]. Days of data collection were reviewed and divided into three recall day patterns: all weekdays; two weekdays and one weekend day; or one weekday and two weekend days. Normality of mReg, ecSI 2.0^TM^ and healthy eating index were assessed with skewness and kurtosis values ≤1 and the linear appearance of the Q–Q plot. Internal consistency of the ecSI 2.0^TM^ was assessed with Cronbach’s alpha. Sample representativeness was assessed with means testing for continuous variables and chi-squared for categorical variables. A Bonferroni correction was applied to multiple contingency tests using chi-squared.

Following the a priori development of mReg scores, associations between mReg scores and HEI total and subcomponent scores were examined with partial correlations, controlling for recall day pattern. mReg scores were examined for association with demographic and culinographic factors using *t*-tests or one-way ANOVA and post hoc Scheffé tests. The HEI of regular and irregular eaters (i.e., those with mReg scores at or above the median vs. <median) was compared with a univariate general linear model controlling for recall day pattern. Linear regression was used to predict HEI from mReg scores and mReg scores from responses to the ecSI 2.0^TM^ item, “I eat regular meals.” Multicollinearity was examined with the variable inflation factor. Test–retest mReg reliability was examined with a paired *t*-test and a repeated measures general linear model controlling for recall day pattern. A paired *t*-test was also used to compare how intervals between first and third recalls in the first dietary assessment differed from that of the retest dietary recalls. Data were analyzed with SPSS 25.0 (IBM, Armonk, NY, USA), *P* values < 0.05 were considered significant.

## 3. Results

### 3.1. Description of Participants

Of 160 who attempted a dietary assessment at baseline or in the longitudinal follow-up, 149 completed 3 dietary recalls with 11 completing only 1 or 2 recalls. Seven parents who completed a dietary assessment had two children in the study and they participated in dietary assessments for both children; only the first dietary assessment was considered for this research question. Only the 142 unique parents (*n* = 67 at baseline of study and *n* = 75 from longitudinal follow-up) completing 3 dietary recalls were included. Of these 142, 28 who had completed a set of three recalls at the baseline of their child’s study participation, completed another set of 3 recalls in the longitudinal follow-up from 2 to 5 years later, depending on the year of their child’s participation in the *FFF* study.

Parents from all four cohorts, all 8 schools, 32 of 40 classrooms, and all 4 parent treatment groups participated, with similar representation in both treatment groups of the prime study. Although 52% were parents of boys, 88% (*n* = 125) of the respondents were female, with a mean age of 38.1 ± 6.2 years and mean BMI of 25.4 ± 5.9. The majority had a normal BMI (*n* = 81; 57%) with less than half overweight (*n* = 39; 28%) or obese (*n* = 21; 15%). This mostly white (*n* = 131; 92%), non-Hispanic (*n* = 131; 92%) sample was highly educated with 46 (34%) reporting a post-graduate degree, 53 (39%) a 4-year college degree and 31 (23%) some post high school/technical/military training. However, 20% (*n* = 29) had participated at some time in the Special Supplemental Nutrition Assistance Program (SNAP), 36 (25%) in the Supplemental Nutrition Assistance Program for Women, Infants, and Children (WIC), and 24 (17%) had utilized food banks/pantries. Nearly one third (*n* = 46) had utilized at least one income-based assistance program and 16% (*n* = 23) reported worrying often or always about having enough money for food, with another 19 (13%) noting worry about this sometimes. Perceived stress was not low with a mean score on the visual analog scale of 6.6 ± 1.9 and a median of 7.0 (maximum 10). Of the types of food preparation listed, 76% (*n* = 108) indicated making homestyle, from scratch foods, 62% (*n* = 88) low-fat “healthy” foods, and 40% (*n* = 57) used speed scratch preparation. About half (50%, *n* = 70) were eating competent. Healthy eating index values were above the 50th percentile, but areas for dietary improvement were evident. Additional characteristics pertinent to food preparation, planning and management practices are in Table 2.

Parents completing three dietary recalls did not differ from parents not participating in dietary recalls or only completing one or two recalls in ethnicity, education level, SNAP or WIC participation, worry about money for food, feelings about cooking, food preparation habits, parent feeding style, perceived stress, eating competence or BMI. Parents completing three dietary recalls were younger at baseline (38.1 ± 6.2 vs. 39.7 ± 5.8; *p* = 0.01; observed power 0.735) than parents with less or no dietary assessment participation (*n* = 288). Demographic and food-related practices did not differ between parents completing one or two dietary recalls and those completing three dietary recalls.

Test–retest participants (*n* = 28), i.e., those completing three 24-h recalls both when their child was in the *FFF* program and in the longitudinal follow-up when the entire study was completed, did not differ from parents completing the three recalls at only one time point for demographic, eating behavior or food preparation/management factors assessed. They represented seven of the eight schools, 17 classrooms, all parent treatment groups and included 14% (*n* = 4) fathers with nearly equal numbers of children in the two treatment groups of the prime study. Time between original baseline dietary assessment and post-intervention retest varied based on cohort participation from three (*n* = 14) to four (*n* = 4) to five (*n* = 10) years. The number of days between recall 1 and recall 3 for their baseline assessment (13.7 ± 9.1; median 10, range 4–34 days) was not significantly different from the recall interval for their follow-up dietary assessment (10.6 ± 7.1, median 8.5, range 4–38 days).

### 3.2. Recall Day Patterns

Dietary intake was provided for two weekdays and one weekend day for 67% (*n* = 95), 2 weekend days and one weekday for 11% (*n* = 15) and three weekdays for 23% (*n* = 32) of study participants. mReg scores differed by recall pattern for each of the three temporally driven mReg versions (Table 3) with highest scores for those recalling only weekday meals. Post hoc tests revealed significant differences between those with three weekday recalls and those with two weekend day recalls, not between either of those with one weekend day recall. The baseline recall pattern of the test-retest participants differed from those who did not participate in the follow-up retest (*p* = 0.005). Their baseline assessment consisted of mostly 2 weekday and 1 weekend assessments (93%, *n* = 26) compared to 61% (*n* = 68) for those not in the test-retest sample. In addition, the recall day pattern differed (*p* = 0.042) between the two time points for the test-retest sample. The pattern was the same between the two measures for only 13 of the 28 test-retest participants with nearly all of the shift being toward recalls from three weekdays. The recall day pattern for the second measure included 39% (*n* = 11) from three weekdays, 42% (*n* = 12) from 2 weekdays and 1 weekend day and 18% (*n* = 5) from 2 weekend days and one weekday.

For those providing all recalls on weekdays (*n* = 32), mReg1 was positively associated with response to the ecSI 2.0^TM^ item “I eat regular meals” (r = 0.36, *p* = 0.045) and response on that item significantly predicted mReg1 score (Intercept 6.48, β 0.67, *p* = 0.045). This relationship was not evident for persons with weekend day recalls.

BMI, compared by frequency of regular breakfasts among those with dietary recalls from at least 2 weekdays (*n* = 127), was significantly (*p* = 0.049) higher for those rarely/never eating breakfast (27.6 ± 1.2, *n* = 47) than those often/sometimes (24.9 ± 1.7, *n* = 16) or always (24.2 ± 0.7, *n* = 64) eating breakfast when controlling for recall pattern. This relationship was not found when including those with two weekend day recalls.

### 3.3. mReg Characteristics and Association with Dietary Quality and BMI

**Figure 2:**
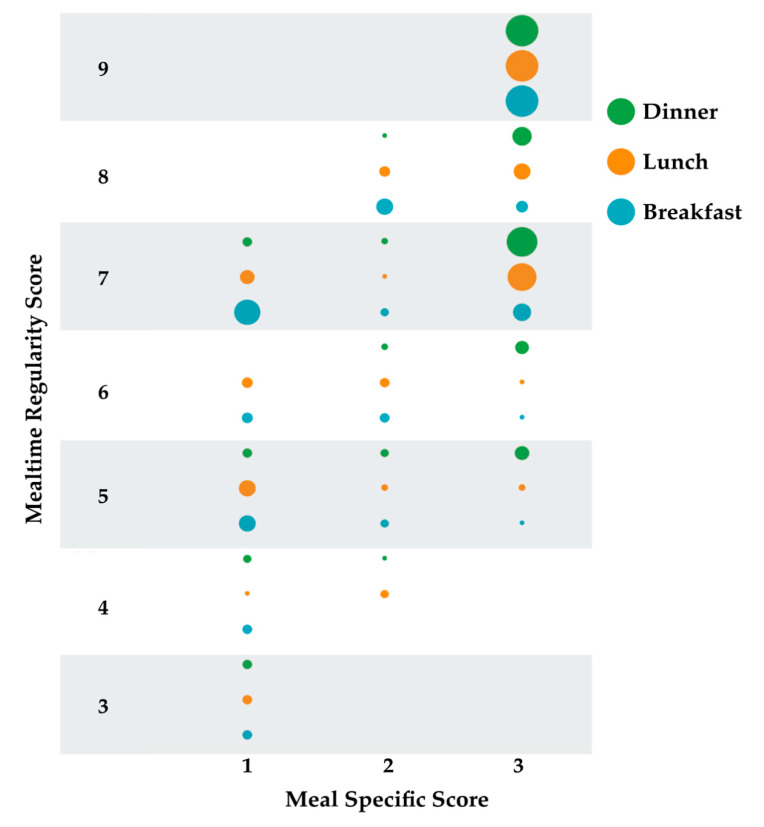
Density distribution of meal-specific scores for each mealtime regularity score. Meal specific scores are 1 rarely/never; 2 often/sometimes; 3 always. Circles represent counts with larger circles indicating more counts. Counts range from 1 [e.g., 1 participant with an mReg1 score of 4 had a score of 1(rarely/never) for lunch; 1 participant with an mReg1 score of 5 had a score of 3 (always) for breakfast] to 45 [All persons with an mReg1 score of 9 had a score of 3 (always) for each meal].

For each definition of mealtime regularity, distribution of descriptor-specific responses revealed greatest differences in frequency of lunch and dinner with greater numbers being categorized as *always* eating lunch and dinner regularly for mReg1 (Table 1). In addition, mean mReg1 scores were higher than mReg2 or mReg3 (Table 1). A cluster graph of the density of meal specific scores (i.e., 3, 2 or 1) demonstrated that breakfast and lunch irregularity contributed equally for mReg1 intermediate scores of 5 and 6, but for the mReg1 score of 7 only breakfast continued to be highly irregular (Figure 2).

The mReg, ecSI 2.0^TM^ and HEI scores were normally distributed. mReg1, but not mReg2 or mReg3 scores, were related to dietary quality, BMI and ecSI 2.0™ subscales. After controlling for pattern of recall days, mReg1 was significantly correlated with total HEI, total fruit and whole fruit components (Table 4). HEI and ecSI2.0^TM^ were significantly correlated (r = 0.26, *p* = 0.002, *n* = 135); mReg1 was associated with HEI even when controlling for ecSI 2.0^TM^ (r = 0.24, *p* = 0.005). mReg2 and mReg3 were associated only with total protein foods (Table 4). mReg1 was significantly inversely associated with BMI (r= −0.18, *p* = 0.036) after correction for day of recall pattern and was associated with better internal regulation (r = 0.19, *p* = 0.032). Body mass index tended (*p* = 0.068) to be lower for participants who always ate breakfast regularly (24.4 ± 5.2; *n* = 67) compared to those eating breakfast with less regularity (26.2 ± 6.4; *n* = 75) when version 1 scoring system was applied.

The mReg scores did not differ by SNAP participation or education level and were not associated with worrying about money for food, how often food is prepared at home, amount of time spent preparing meals or parent feeding style. Those with mReg scores at or above the median were categorized as *regular eaters* (*n* = 62) and as *irregular eaters* (*n* = 80) for scores below the median. Even when controlling for recall day pattern, regular eaters as defined by mReg1, but not mReg2 or mReg3, had significantly higher HEI scores (*p* = 0.027; 62.5 (SEM 2.4) vs. 55.4 (SEM 2.0)). Additionally, unlike mReg2 and mReg3, mReg1 significantly predicted total HEI scores (*p* = 0.001; β 2.265, intercept 42.199).

The breakfast specific regularity value (1–3 possible) for mReg1 was examined for its contribution to the total mReg1 score revealing a widely ranging pattern of breakfast consumption. For 32%, breakfast regularity contributed to <one-fourth of mReg1 but contributed one-fourth through one-third of the mReg1 score for 50% of participants. Only 18% of participants had breakfast regularity values that contributed more than one-third of the mReg1 score.

### 3.4. Test–Retest Reliability

Test–retest reliability was affirmed. Initial and follow-up mReg scores were not significantly different with a paired *t*-test (mReg1 7.3 ± 1.3, 7.3 ± 1.8; mReg2 5.4 ± 1.3, 5.4 ± 1.6; mReg3 5.5 ± 1.3, 5.4 ± 1.7; all *n* = 28) or after controlling for recall day pattern using a repeated measures GLM.

## 4. Discussion

It is both interesting and baffling that timing of food intake, fundamental to human health and disease, has had limited scientific analysis. The meaning of the term *regular meals* remains under scrutiny, but this study offers a practical, temporally based suggestion aligned with dietary quality.

Food intake was measured and categorized in many ways, e.g., number of meals/day, number of meals/week, time between meals in a day, usual time of day for meals, caloric distribution each day and location of meals. Most of these paradigms do not account for shift work, global travel, cultural differences in defining meals and/or snacks and differences in preparation and expected portion size of the same foods [22]. Thus, inventive measures of mealtime regularity have been developed. The chaotic eating index compares self-defined 30-min potential blocks of eating times to self-reported number of eating occasions. When using this index, BMI was unrelated to mealtime regularity [25]. Breakfast regularity was examined by comparing intake of persons who ate breakfast on one recall day with their intake on another day that breakfast was skipped. Findings demonstrated no impact on timing or composition of dinner, some adjustment of timing of lunch and that intake did not compensate completely for the missed breakfast [36]. Another example is classifying eating episodes based on food categories of similar food types and nutrients. In this way, reporting frequency and timing of eating events that include the food-based classifications of eating episodes can reflect on dietary quality and be compared across studies. Specific combinations of food categories are translated to eating occasions, e.g., complete meal, mixed-quality snack, vegetarian meal. This method has an advantage of accommodating shift workers and various dietary patterns but may not translate internationally [26]. Given the rigorous research showing a strong influence of the 24-h circadian rhythm on many physiological processes that influence health and behavior [14,15,19], a definition of mealtime regularity must attend to consistency from day-to-day of times of each eating occasion.

A review of published literature did not reveal a measure of mealtime regularity based on meal-specific day-to-day time of intake across three non-contiguous days. Thus, the lack of “consistent direction from previous research or a consensus about appropriate critical cut points” [24] prompted construction of a novel measure of mealtime regularity (mReg). The mReg defines mealtime regularity by dietary quality and was developed by comparing the time of day 142 adults each consumed breakfast, lunch and dinner across three non-contiguous days. Time of consumption of the participant-defined meals and HEI values were derived from three 24-h recalls using the ASA24 online dietary assessment platform. The resulting measure of mealtime regularity, mReg1, equated strong mealtime regularity with consumption of either breakfasts, lunches or dinners that were 0–60 min apart from the time they were eaten on days and also with lunches or dinners that had one day-to-day consumption interval of up to 120 min provided the meals on the other two days were ≤60 min apart. This measure was associated with total HEI and whole fruit and total fruit subcomponents and tended to associate with total protein and seafood and plant protein subcomponents. Although the sample, which consisted of parents with youth participating in a 4th grade culinary and physical activity intervention, were from only one metropolitan area in one state, they were similar in age, BMI, ethnicity, sex, education level, food preparation practices, worry about money for food, and ecSI 2.0^TM^ scores to a larger sample (*n* = 832) from 33 states with approximately 50% being parents [34]. Dietary quality was higher in this sample than the validation sample from the NHANES study of Americans two years of age in 2003–2004 [35]. Greatest discrepancies were for greens and beans, total vegetable and whole grain subcomponents. Total HEI score was approximately nine percentile points above the validation sample, but <6 percentile points higher for women. Although total fruit, whole fruit, and total protein values were identical or nearly so, further study with a sample that has a lower dietary quality would be prudent.

The mReg values derived from more narrowly defined day-to-day mealtime differences intervals were not correlated with these measures of dietary quality. The reasons for the lack of association of dietary quality with the more rigid measures of mealtime regularity (i.e., mReg2 and mReg 3) are purely speculative. Perhaps participants who needed to eat within a narrow window of time each day held more than one job or worked in factories or businesses with limited individual flexibility and demands that interfered with dietary quality, e.g., time to prepare homestyle meals. Participants who ate within a narrow window of time may also have restricted their intake in quantity or variety, leading to lower HEI scores. Follow-up qualitative studies may identify lifestyle characteristics and eating behaviors that better inform the rationale for a more flexible definition of regular meals.

The use of dietary quality as an arbiter of mealtime regularity was chosen because previous studies have suggested higher dietary quality with “regular” eating, especially breakfast [22,36,37], but mainly because of the recognition more than two decades ago that a measure of mealtime regularity needed to be tested against overall dietary quality [26] and that the need to examine how meal timing is related to dietary quality continues to be recommended [22]. Health status is also a potential comparator against a mealtime regularity index, especially given the newly proposed relationships between microbiota, single nucleotide polymorphisms of several genes and cellular circadian rhythms [14,15,18,19,20]. The only bioclinical measure in this study was self-reported height and weight from which BMI was calculated. Additional examination of mReg1 performance against bioclinical parameters such as HgA1c, measures of oxidative stress, and cardiometabolic biomarkers is suggested.

A higher BMI was associated with lower mealtime regularity (for mReg1) and rarely or never eating breakfast was associated with a higher BMI among those completing at least two of their three recalls on a weekday. This relationship with BMI and mealtime regularity is similar to the findings of Kant and Graubard [36] when they examined breakfast regularity. However, BMI was not associated with a measure of chaotic eating in a large sample from the United Kingdom National Diet and Nutrition Survey [25] as well as in a sample living in the Republic of Ireland who were deemed regular breakfast eaters because they ate breakfast three or four out of four days [37]. A possible confounder offered by those researchers was that overweight persons may have been skipping breakfast in an attempt to lose weight.

However, the reliance on breakfast regularity to indicate BMI or diet quality may not be sound. In this study, breakfast contributed <25% to the mReg1 score for nearly ⅓ of our sample and contributed ⅓ or less to the mReg score for 82% of the sample. When including those with at least two weekday recalls, BMI was higher for those who rarely or never ate breakfast, but not when participants who completed at least two recalls on a weekend day were included. Studies of dietary intake or timing rarely report day of week that dietary data are collected.

### Strengths and Limitations

Several strengths lend credence to the use of mReg1 to define mealtime regularity. Data were collected using the tested ASA24 platform that included dietary data entry tutoring for all participants. Day of week was considered in the analyses and controlled for when comparing mReg to dietary and BMI parameters. The fact that mReg scores differed by number of weekend day recalls supports reporting accuracy and mReg agreement. Despite the variation in day of week recall patterns, test-retest reliability was affirmed when controlling for recall pattern.

Methods employed to assure accurate and consistent meal characterization and to calculate meal-specific intervals from day-to-day were examined independently by more than one author and agreed upon prior to analyses. Foods, nutrient value and pattern on other recalls days were all integrated to clarify mealtimes for the few instances when participants entered confusing information. Response fidelity was evident. For example, a high percentage admitted to skipping breakfast and the response to the item “I eat regular meals” was able to predict mReg1 when considering those with recalls that included <two weekend days.

Several limitations temper the recommendation to define mealtime regularity by the mReg1 intervals based on day-to-day consistency in eating occasions. The sample consisted only of parents of 4th grade youth from one geographic location, limiting the generalizability of these findings. All dietary intake data and height and weight are self-reported and subject to error or purposeful misinformation. However, the ASA24 has undergone significant evaluation and testing for validity [38]. In addition, as part of the *Fuel for Fun* primary study, parents provided height and weight on more than one occasion and a review of BMI changes revealed little to no change over time (data not shown), suggesting that height and weight information were at least consistent, and therefore more likely to be closer to actual values. Self-reported and measured height and weight in young adults have shown moderate to high agreement, supporting the use of online self-reported height and weight [39]. However, greater discrepancies for overweight and overweight adults suggest that future studies of the mReg1 definition of mealtime regularity include measured height and weight [40].

Three 24-h recalls is a standard in dietary intake methodology to assess energy intake [41] but may not be enough to ascertain day-to-day consistency of times of eating occasions suggesting exploration of the mReg1 definition with 24 h recalls from more than three days. An additional limitation was related to the lack of knowledge about participant lifestyle and health information. For example, history of diabetes, gluten enteropathy, allergies or bariatric surgery was unknown as was shift work or vacation status, limiting interpretation of findings. Future studies will want to learn more about participant health and work histories to develop more confidence in this definition of mealtime regularity.

## 5. Conclusions

In alignment with attention to cellular circadian rhythms as markers of health, mealtime regularity was defined according to the day-to-day consistency of eating occasions for breakfast, lunch and dinner, thus reflecting a circadian rhythm of intake. The definition of mealtime regularity most consistent with dietary quality and lower BMI denotes always being consistent as eating each meal within 0 to 60 min of the same time each day, with an option for one day’s lunch or dinner to differ by as much as 120 min as long as the other day differences are 60 min or less. This definition represents a novel approach to defining “regular meals” and has the potential for informing future dietary recommendations.

## Figures and Tables

**Table 1:** Mealtime regularity scoring protocol and outcomes for three meal and temporally specific versions based on three 24-h dietary recalls of parents with children participating as 4th graders in *Fuel for Fun* (*n* = 142).

Version	Meal	Frequency	Score	Frequency-Driven Description of Mealtime Intervals	Frequency *n* (%)	Mealtime Regularity Score
One (1)	Breakfast	always	3	0–60 min between the three breakfasts, no exceptions. *Cannot skip breakfasts* and *get this score.*	67 (47)	Mean (SD) 7.26 (1.60) Median 7.00 Range 3–9 Score Frequency 3 4 4 4 5 16 6 10 7 46 8 17 9 45
		often/sometimes	2	0–90 min between the three breakfasts and no breakfasts skipped **OR** ≤60 min between two breakfasts and skipped one breakfast	21 (15)	
		rarely/never	1	60–90 min between two breakfasts, but the third breakfast was >90 min from the other two **OR** was skipped; **OR** >90 min between two or more breakfasts (regardless of what the third breakfast was); **OR** two or more breakfasts were skipped.	54 (38)	
	Lunch	always	3	0–60 min between all lunches **OR** ≤ 60 min between two lunches and ≤120 min for the third meal. *Cannot skip lunches* and *get this score.*	95 (67)	
		often/sometimes	2	Two lunches were 0–60 min apart, but one lunch was skipped **OR** 60–120 min between two or more lunches with no lunches skipped.	15 (11)	
		rarely/never	1	60–120 min between two lunches, but one lunch was > 120 min from the others or was skipped; **OR** if any lunches were >120 min apart from the rest; **OR** if ≤ two lunches were skipped.	32 (23)	
	Dinner	always	3	0–60 min between all dinners **OR** ≤ 60 min between two dinners and ≤120 min for the third meal. *Cannot skip dinners* and *get this score.*	118 (83)	
		often/sometimes	2	Two dinners were 0–60 min apart, but one dinner was skipped **OR** 60–120 min between ≥ two dinners with no meals skipped.	9 (6)	
		rarely/never	1	60–120 min between two dinners, but one dinner was > 120 min from the others or was skipped; **OR** if any dinners were >120 min apart from the rest; **OR** if ≥ two dinners were skipped.	15 (11)	
Two (2)	Breakfast	always	3	0–30 min between all breakfasts. *Cannot skip breakfasts* and *get this score.*	37 (26)	Mean (SD) 5.54 (1.74) Median 6.00 Range 3–9 Score Frequency 3 21 4 28 5 21 6 23 7 30 8 13 9 6
		often/sometimes	2	0–60 min between all breakfasts and no breakfasts were skipped; **OR** 0–30 min between two breakfasts and one breakfast skipped.	35 (25)	
		rarely/never	1	One breakfast is >60 min from the rest **OR** if 0–60 min between two breakfasts and a third breakfast was skipped; **OR** if ≥ two breakfasts were skipped.	70 (49)	
	Lunch	always	3	0–30 min between all lunches. *Cannot skip lunches* and *get this score.*	47 (33)	
		often/sometimes	2	0–60 min between all lunches and no lunches were skipped; **OR** 0–30 min between two lunches and one lunch was skipped.	38 (27)	
		rarely/never	1	One lunch is > 60 min from the rest **OR** if 0–60 min between two lunches and a third lunch was skipped; **OR** if ≥ two lunches were skipped.	57 (40)	
	Dinner	always	3	0–30 min between all dinners. *Cannot skip dinners* and *get this score.*	42 (30)	
		often/sometimes	2	0–60 min between all dinners and no dinners were skipped; **OR** 0–30 min between two dinners and one dinner was skipped.	35 (25)	
		rarely/never	1	One dinner is >60 min from the rest **OR** if 0–60 min between two dinners and a third dinner was skipped; **OR** if ≥ two dinners were skipped.	65 (46)	
Three (3)	Breakfast	always	3	0–45 min between all breakfasts. *Cannot skip breakfasts* and *get this score*	42 (30)	Mean (SD) 5.57 (1.74) Median 6.00 Range 3–9 Score Frequency 3 21 4 26 5 22 6 23 7 30 8 14 9 6
		often/sometimes	2	0–60 min between all breakfasts and no breakfasts were skipped; **OR** 0–45 min between two breakfasts and one breakfast skipped.	30 (21)	
		rarely/never	1	If any meal is more than 60 min from the other two meals (skipping is not applied here); **OR** if 0–60 min between two meals, but one was skipped; **OR** if two or more meals were skipped.	70 (49)	
	Lunch	always	3	0–30 min between all lunches. *Cannot skip lunches* and *get this score.*	47 (33)	
		often/sometimes	2	0–60 min between all lunches and no lunches were skipped; **OR** 0–30 min between two lunches and one lunch was skipped	38 (27)	
		rarely/never	1	One lunch is >60 min from the rest **OR** if 0–60 min between two lunches and a third lunch was skipped; **OR** if ≥ two lunches were skipped.	57 (40)	
	Dinner	always	3	0–30 min between all dinners. *Cannot skip dinners* and *get this score.*	42 (30)	
		often/sometimes	2	0–60 min between all dinners and no dinners were skipped; **OR** 0–30 min between two dinners and one dinner was skipped.	35 (25)	
		rarely/never	1	One dinner is >60 min from the rest **OR** if 0–60 min between two dinners and a third dinner was skipped; **OR** if ≥ two dinners were skipped.	65 (46)	

**Table 2:** Culinographic, eating behavior and dietary intake characteristics of parents of 4th grade youth participating in a dietary assessment component of a nutrition education intervention ^1^.

Plans meals ahead of time	
Never	1 (1)
Seldom	5 (4)
Sometimes	44 (31)
Most of the time	66 (47)
Almost always	26 (18)
Amount of time spent preparing a meal	
Do not prepare own meals	2 (1)
<15 min	4 (3)
15–45 min	121 (85)
>45 min	15 (11)
Number of times/week prepared food at home	
1–3 times	7 (5)
4–6 times	74 (52)
Every day	61 (43)
Reports eating regular meals	
Rarely/never	2 (1)
Sometimes	12 (9)
Often	54 (38)
Always	73 (52)
Reports eating a variety of foods	
Rarely/never	5 (4)
Sometimes	28 (20)
Often	67 (48)
Always	41 (29)
Feeling toward cooking	
I like to cook	72 (51)
I do not mind cooking	54 (38)
I do not like to cook	15 (11)
Frequency of worrying about money for food	
Never	46 (32)
Rarely	54 (38)
Sometimes	19 (13)
Often	12 (9)
Always	11 (8)
Parent feeding style ^2^	
Uninvolved	29 (22)
Indulgent	40 (30)
Authoritarian	40 (30)
Authoritative	23 (17)
Eating competence (ecSI 2.0^TM^ scores) ^3,4^	
Total score (*n* = 135) Subscales Eating attitudes (*n* = 141)	32.4 ± 8.1 (6–48) 12.9 ± 3.6 (0–18)
Internal regulation (*n* = 141)	3.9 ± 1.4 (0–6)
Food acceptance (*n* = 139)	5.3 ± 2.0 (1–9)
Contextual skills (*n* = 139)	10.4 ± 2.8 (2–15)
Healthy eating index ^4,5^	
Total	58.6 ± 13.1. (22.0–89.1)
Energy (Kcal)	1818.2 ± 573.7 (608.2–4422.5)
Total vegetable	4.1 ± 1.2 (0.8–5.0)
Green beans	3.0 ± 2.2 (0–5)
Total fruit	2.7 ± 1.9 (0–5)
Whole fruit	3.1 ± 2.1 (0–5)
Whole grain	3.1 ± 3.0 (0–10)
Refined grain	6.9 ± 3.0 (0–10)
Dairy	6.5 ± 2.8 (0–10)
Total protein foods	4.6 ± 0.8 (1.2–5)
Seafood and plants	3.7 ± 1.8 (0–5)
Sodium	2.9 ± 2.8 (0–10)
Fatty acids	4.5 ± 3.1 (0–10)

^1^ Unless specified *n* = 142; table entries are frequencies (%). ^2^ Measured with caregiver’s feeding style questionnaire; *n* = 132; ^3^ Measured with the Satter Eating Competence Inventory (ecSI 2.0^TM^); ^4^ Table entries are mean ± SD (range). ^5^ Possible ranges are 0–100 (Total); 0–10 (Whole grain, Refined grain, Dairy, Sodium, Fatty Acids; 0–5 (Total Vegetable, Green Beans, total fruit, whole fruit).

**Table 3:** Mealtime regularity score compared among day of week recall patterns.

Recall Pattern	Mealtime Regularity Score ^1^		
	Version 1	Version 2	Version 3
Three weekdays (*n* = 32)	8.03 (1.47)	6.44 (1.70)	6.50 (1.67)
Two weekdays/1 weekend day (*n* = 95)	7.15 (1.52)	5.29 (1.68)	5.33 (1.69)
1 weekday/2 weekend days (*n* = 15)	6.33 (1.76)	5.13 (1.64)	5.13 (1.64)
*p* ^2^	0.001	0.003	0.002
	**Retest Mealtime Regularity Scores**		
Three weekdays (*n* = 11)	8.27 (0.79)	6.09 (1.70)	6.18 (1.72)
Two weekdays/1 weekend day (*n* = 12)	6.75 (2.26)	4.92 (1.56)	5.00 (1.65)
1 weekday/2 weekend days (*n* = 5)	6.20 (1.30)	4.80 (1.30)	4.80 (1.30)
*p*	0.042	NS	NS

^1^ Possible score 3–9; Table entries are means (SD); ^2^ Observed power was 0.9 for each version comparison; NS: non-significant.

**Table 4:** Correlation between mealtime regularity scores and healthy eating index scores controlled for pattern of recall days among 142 parents of 4th grade children participating in a culinary and physical activity program ^1^.

Healthy Eating Index ^3^	Mealtime Regularity Score ^2^		
	Version 1	Version 2	Version 3
Total score	0.26; 0.002	0.12; NS	0.12; NS
Energy (Kcal)	0.03; NS	−0.04; NS	−0.04; NS
Total vegetables	0.04; NS	−0.03; NS	−0.02; NS
Green beans	0.06; NS	−0.12; NS	−0.11; NS
Total fruit	0.18; 0.035	0.12; NS	0.13; NS
Whole fruit	0.17; 0.040	0.09; NS	0.10; NS
Whole grain	0.13; NS	0.009; NS	0.001; NS
Dairy	0.02; NS	0.09; NS	0.09; NS
Total protein	0.15; NS ^4^	0.19; 0.027	0.18; 0.03
Seafood plant	0.15; NS ^4^	0.16; NS ^4^	0.15; NS ^4^
Refined grain	0.06; NS	−0.03; NS	−0.03; NS
Sodium	0.03; NS	−0.05; NS	−0.05; NS
Fatty acids	0.12; NS	0.07; NS	0.07; NS

^1^ Table entries are Pearson’s correlation coefficient; 2-tailed *p*-values; ^2^ possible range 3 (no/low regular meals) to 9 (highly regular meals); ^3^ based on healthy eating index 2010; ^4^ *p* = 0.07; NS: non-significant.
